# Prevalence of Type 2 Diabetes in South Africa: A Systematic Review and Meta-Analysis

**DOI:** 10.3390/ijerph18115868

**Published:** 2021-05-30

**Authors:** Carmen Pheiffer, Victoria Pillay-van Wyk, Eunice Turawa, Naomi Levitt, Andre P. Kengne, Debbie Bradshaw

**Affiliations:** 1 Biomedical Research and Innovation Platform, South African Medical Research Council, Tygerberg 7505, South Africa; 2 Division of Medical Physiology, University of Stellenbosch, Tygerberg 7505, South Africa; 3 Department of Obstetrics and Gynaecology, University of Pretoria, Pretoria 0001, South Africa; 4 Burden of Disease Research Unit, South African Medical Research Council, Tygerberg 7505, South Africa; Victoria.Pillay-vanwyk@mrc.ac.za (V.P.-v.W.); Eunice.Turawa@mrc.ac.za (E.T.); Debbie.Bradshaw@mrc.ac.za (D.B.); 5 Department of Medicine, Division of Endocrinology, University of Cape Town, Observatory 7925, South Africa; Naomi.Levitt@uct.ac.za; 6 Non-Communicable Diseases Research Unit, South African Medical Research Council, Tygerberg 7505, South Africa; Andre.Kengne@mrc.ac.za; 7 School of Public Health and Family Medicine, University of Cape Town, Observatory 7925, South Africa

**Keywords:** prevalence, South Africa, type 2 diabetes mellitus, impaired glucose tolerance, impaired fasting glucose, newly diagnosed diabetes

## Abstract

Synthesis of existing prevalence data using rigorous systematic review methods is considered an effective strategy to generate representative and robust prevalence figures to inform health planning and policy. The purpose of this systematic review was to identify, collate, and synthesise all studies reporting the prevalence of total and newly diagnosed type 2 diabetes (T2DM), impaired glucose tolerance (IGT), and impaired fasting glucose (IFG) in South Africa. Four databases, PubMed, Scopus, Web of Science, and African Index Medicus were searched for articles published between January 1997 and June 2020. A total of 1886 articles were identified, of which 11 were included in the meta-analysis. The pooled prevalence in individuals 25 years and older was 15.25% (11.07–19.95%) for T2DM, 9.59% (5.82–14.17%) for IGT, 3.55% (0.38–9.61%) for IFG, and 8.29% (4.97–12.34%) for newly diagnosed T2DM. Although our pooled estimate may be imprecise due to significant heterogeneity across studies with regard to population group, age, gender, setting, diagnostic test, and study design, we provide evidence that the burden of glucose intolerance in South Africa is high. These factors contribute to the paucity of representative T2DM prevalence data. There is a need for well-designed epidemiological studies that use best-practice and standardised methods to assess prevalence.

## 1. Introduction

Diabetes mellitus is an escalating public health crisis and among the top ten leading causes of death worldwide [1]. The condition affects approximately 451 million adults worldwide, with projections of 693 million cases by 2045 [2]. The largest relative increase is predicted for Africa, where in 2017, 15.5 million adults had diabetes, with 69.2% of people unaware of their diabetic status. Africa is already grappling with high rates of communicable diseases such as human immunodeficiency virus (HIV)/acquired immunodeficiency syndrome (AIDS) and tuberculosis (TB) [3], thus diabetes poses a serious health and economic burden to these already overburdened and under-resourced health systems. To achieve the United Nations’ Sustainable Development Goal 3.4, which aims to reduce premature mortality from non-communicable diseases (NCDs) by a third by 2030 [4], requires urgent action and effective intervention strategies to combat the rising diabetes epidemic in Africa.

South Africa is ranked as an upper-middle-income country [5] and is the second largest economy in Africa. Despite this, it is plagued by high economic and health inequalities due to years of racial and gender discriminatory policies, which have led to a suboptimal health system, with health outcomes often worse than those in poorer countries [6]. South Africa has a unique quadruple disease burden characterised by high rates of HIV/AIDS and TB, non-communicable diseases, maternal and childhood mortality, and injury-related disorders [3]. The prevalence of T2DM has almost doubled from 5.5% in 2000 to 9% in 2009 [7,8], and although these estimates were based on robust modelling methods, they probably underestimate the current disease burden, as risk factors for T2DM have significantly increased in recent years. Rapid urbanisation characterised by the adoption of unhealthy energy-dense diets and physical inactivity have contributed to the steadily increasing obesity epidemic, with 69% of women and 39% of men in South Africa being overweight or obese [9]. Obesity is a major contributor to the T2DM epidemic, with excess bodyweight estimated to account for 87% of T2DM cases in South Africa [10]. Recent studies estimate the T2DM age-adjusted prevalence at 13.1% [11] or 26.3% [12] depending on population group. In addition, these studies report high rates of newly diagnosed diabetes [11,12] and glucose intolerance, which is associated with a 5–12 times higher annual risk of developing T2DM [13]. Together, these studies demonstrate a high overall burden of dysglycaemia in South Africa.

Reliable national epidemiological data on T2DM prevalence are required to inform health policy and planning. Although several studies have measured the prevalence of T2DM in South Africa [11,12,14,15,16,17,18,19], they are generally too small to individually give generalisable prevalence data, while the methodological concerns (sub-optimal response rates and sensitivity of diagnostic tests) of the two national surveys [20,21], prompted this review. Pooling and synthesis of existing prevalence data using rigorous systematic review methods is considered an effective strategy to generate representative and robust prevalence figures to inform health planning and policy [22,23]. This review explores the availability and quality of T2DM prevalence data in South Africa. Studies reporting the prevalence of total and newly diagnosed T2DM, impaired glucose tolerance (IGT), and impaired fasting glucose (IFG) between January 1997 and June 2020 were identified, collated, and synthesised using rigorous systematic review methods and standardised risk-of-bias tools.

## 2. Methods

This systematic review was conducted adhering to the published protocol [24] and is registered with the Prospective Register of Systemic Reviews (PROSPERO): CRD42017071280. The review adheres to the Preferred Reporting Items for Systematic Reviews and Meta-Analyses (PRISMA) guidelines (Table S1) [25].

### 2.1. Literature Search

Four major databases (PubMed, Scopus, Web of Science, and African Index Medicus) were searched for studies on the prevalence of T2DM in South Africa, published between January 1997 and June 2020. Search terms included keywords and medical subject headings (MeSH) such as diabetes mellitus, T2DM, hyperglycaemia, blood glucose, glycosylated haemoglobin, diagnosis, IGT, IFG, and undiagnosed diabetes—including corresponding synonyms and associated terms for each item. The search strategy is shown in Table S2 and was adapted for each database. Reference lists of eligible studies were searched to identify studies for possible inclusion, and we contacted experts in the field to identify further potentially eligible studies.

### 2.2. Inclusion and Exclusion Criteria

Population-based cross-sectional studies were included if they were conducted in South Africa and had more than 100 participants regardless of gender, population group, age, and socioeconomic and educational background, and reported the primary outcome (T2DM prevalence) according to World Health Organization (WHO) diagnostic criteria [26,27,28]. Population group was classified according to previously defined apartheid racial categories of Black African, Indian, White (European descent), and Coloured (of mixed ancestry according to the preceding categories), which was introduced into the new birth and death notifications in 1998 to track health inequalities [29]. Only individuals aged 25 years and older were included.

### 2.3. Outcome Measures

#### 2.3.1. Primary Outcome

T2DM is defined as fasting plasma glucose (FPG) ≥ 7.0 mmol/L, 2-h oral glucose tolerance test (OGTT) plasma glucose ≥ 11.1 mmol/L, glycated haemoglobin (HbA1c) ≥ 6.5% (48 mmol/mol), or self-reported use of diabetes drugs.

#### 2.3.2. Secondary Outcomes

IGT (FPG < 7.0 mmol/L and 2-h OGTT plasma ≥ 7.8 mmol/L and < 11.1 mmol/L); IFG (FPG > 6.1 mmol/L and < 7.0 mmol/L); and newly diagnosed T2DM are defined as the number of new cases of diabetes as a proportion of the sample size [26,27,28].

### 2.4. Study Selection, Quality Assessment and Data Extraction

After removal of duplicates, two reviewers (C.P. and V.P.-v.W. or E.T.) independently screened titles and abstracts to select full-text articles for inclusion. The two reviewers assessed each included study for risk of bias using a web-based standardised checklist for systematic review of observational epidemiological studies, Burden of Disease Review Manager (BODRevMan), developed by the Burden of Disease Research Unit of the South African Medical Research Council [30], that was adapted from the risk-of-bias tool for population-based studies [31] and the Newcastle–Ottawa Scale for assessing the quality of non-randomised studies [32,33]. BODRevMan contains eight scoring domains to assess internal (case definition, data collection, uncertainty of estimation, appropriateness of time factor for outcome measure, appropriateness of numerator and denominator in calculation of estimate, and confounding factors) and external (representativeness and non-response bias) validity, with an overall quality score of 20 (Table S3). A study is classified as having a low risk of bias (14 to 20), moderate (7 to 13), or high risk of bias (1 to 6) based on the total score. Two authors independently extracted and recorded data in BODRevMan. Data extracted included date of publication and details of study design, location, population characteristics, response rates, T2DM, IGT, IFG and newly diagnosed T2DM prevalence, and case definition as reported in the study. When possible, crude prevalence estimates were recalculated in people aged 25 years and older using the number of cases as numerator and sample size as denominator. For national surveys (South African National Health and Nutrition Examination Survey (SANHANES) and South African Demographic Health Survey (SADHS)), data were reanalysed taking design effect into consideration. Comorbid disease (HIV/AIDS and TB) was documented when reported. Corresponding authors were contacted when further clarification/more information was required. Disagreements or uncertainties at each stage of the review process (screening, risk-of-bias assessment, and data extraction) were resolved by discussion and consensus between the two reviewers, or with a third reviewer if disagreement persisted (C.P. and V.P.v.-W. or E.T.).

### 2.5. Data Synthesis and Analysis

Prevalence data from individual studies were pooled using the statistical software STATA v14 (StataCorp, College Station, TX, USA) and the metaprop package [34]. Metaprop models the prevalence estimates using the exact binomial distribution and then applies the Freeman–Turkey double arcsine variance stabilising transformations to stabilise variances before pooling, and then back-transforms the estimates. The pooled estimates are then computed using the procedure described by DerSimonian and Laird [35]. The magnitude of heterogeneity between studies was assessed using the I^2^ index, while subgroup analysis and meta-regression using the metareg command in STATA was conducted to identify potential sources of heterogeneity. The symmetry of funnel plots were visually inspected to assess publication bias, while the Egger [36] and Beggs [37] tests were conducted to statistically assess publication bias [36]. Results are displayed using tables and forest plots as appropriate.

### 2.6. Confidence in Cumulative Evidence

The strength of evidence was assessed using the Grading of Recommendations Assessment, Development, and Evaluation (GRADE) method [38], which scores the quality of the studies and the strength of the evidence as very low, low, moderate, or high based on methodological flaws within the included studies, consistency of results across diverse studies, precision of estimates, and publication bias [39,40].

### 2.7. Patient and Public Involvement

Patients and the public were not involved in the study.

## 3. Results

### 3.1. Results of Study Search

**Figure 1:**
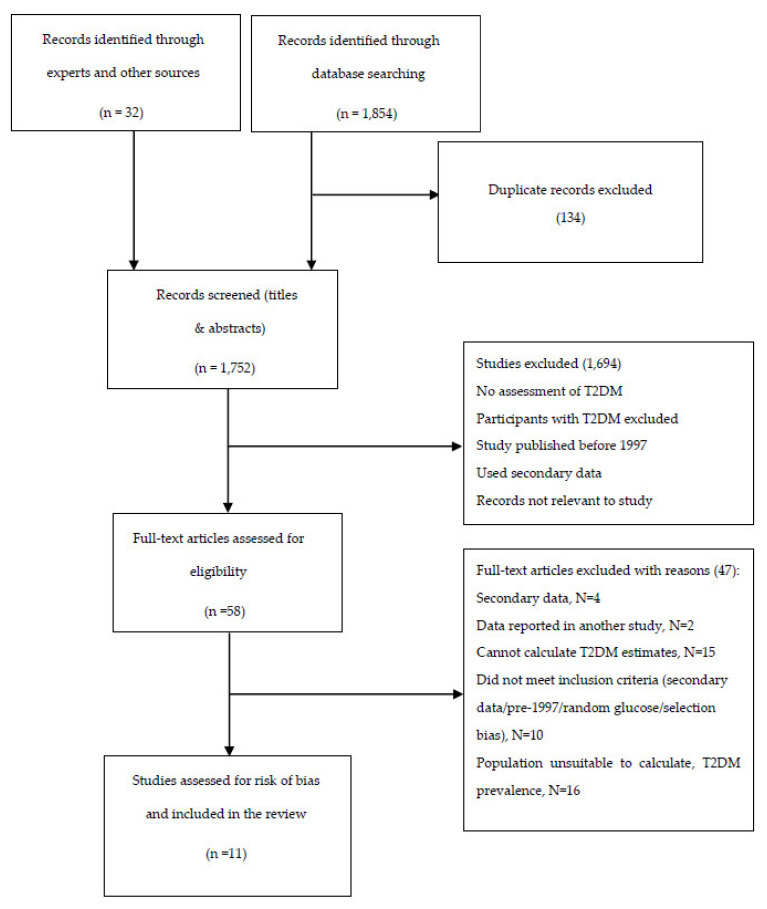
Flow diagram showing selection of studies for inclusion in the systematic review.

A total of 1886 articles were identified with 1752 records left after removal of duplicates. Titles and abstracts were screened for eligibility; from the 56 selected for full-text review, 11 studies met the inclusion criteria and were included in the systematic review. Figure 1 displays the flow diagram for the review process.

### 3.2. Characteristics of Included Studies

Eleven studies were included, which comprised two national surveys SANHANES and SADHS, and nine community-based studies. Studies were published between 2001 and 2019 and were conducted between 1997 and 2016. SANHANES and SADHS were conducted across different provinces, geographical locations (urban vs. rural), and population groups as defined by South African birth and death notification registries [29]. The majority of the community-based surveys were conducted in the Western Cape (WC, four), followed by KwaZulu Natal (KZN, three), and one each in Limpopo and Free State (FS). One study was conducted in both an urban and rural setting, five in urban settings only and three in rural populations only. Four studies were conducted in Black Africans, followed by three in Coloureds, one in Indians, and one in a mixed population of both Black Africans and Coloureds. In many studies methodological reporting was suboptimal, which limited the risk-of-bias assessment.

### 3.3. Type 2 Diabetes Prevalence

**Figure 2:**
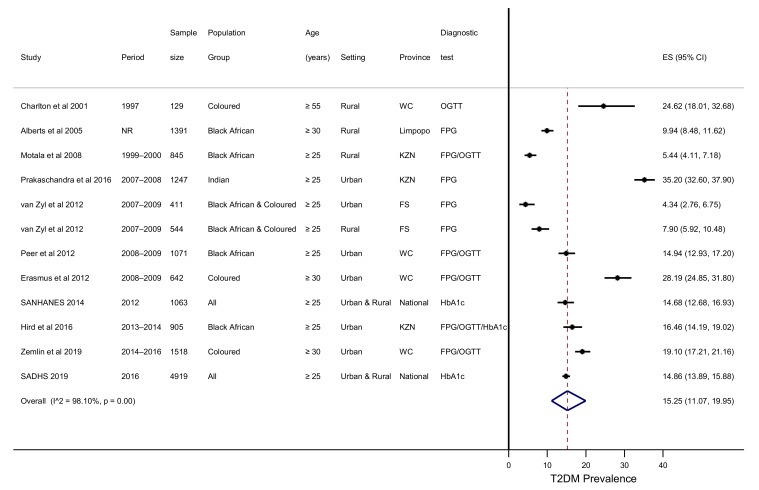
Forest plot of T2DM prevalence. Data are represented as the crude prevalence and 95% CI. EC, Eastern Cape; KZN, KwaZulu Natal; WC, Western Cape. NR, Not Reported.

The pooled prevalence of T2DM was 15.25% (11.07–19.95%), with significant heterogeneity observed between studies (I^2^ = 98.10%, *p* < 0.001) (Table 1, Figure 2). The characteristics of the included studies are listed in Table S4. Subgroup analysis and meta-regression for population characteristics, geographic location, and diagnostic test did not explain heterogeneity across studies.

### 3.4. T2DM Prevalence by Subgroups

**Figure 3:**
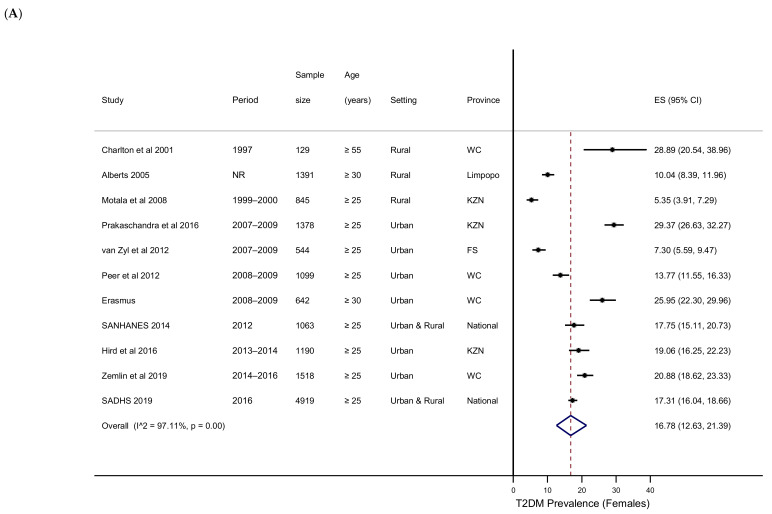

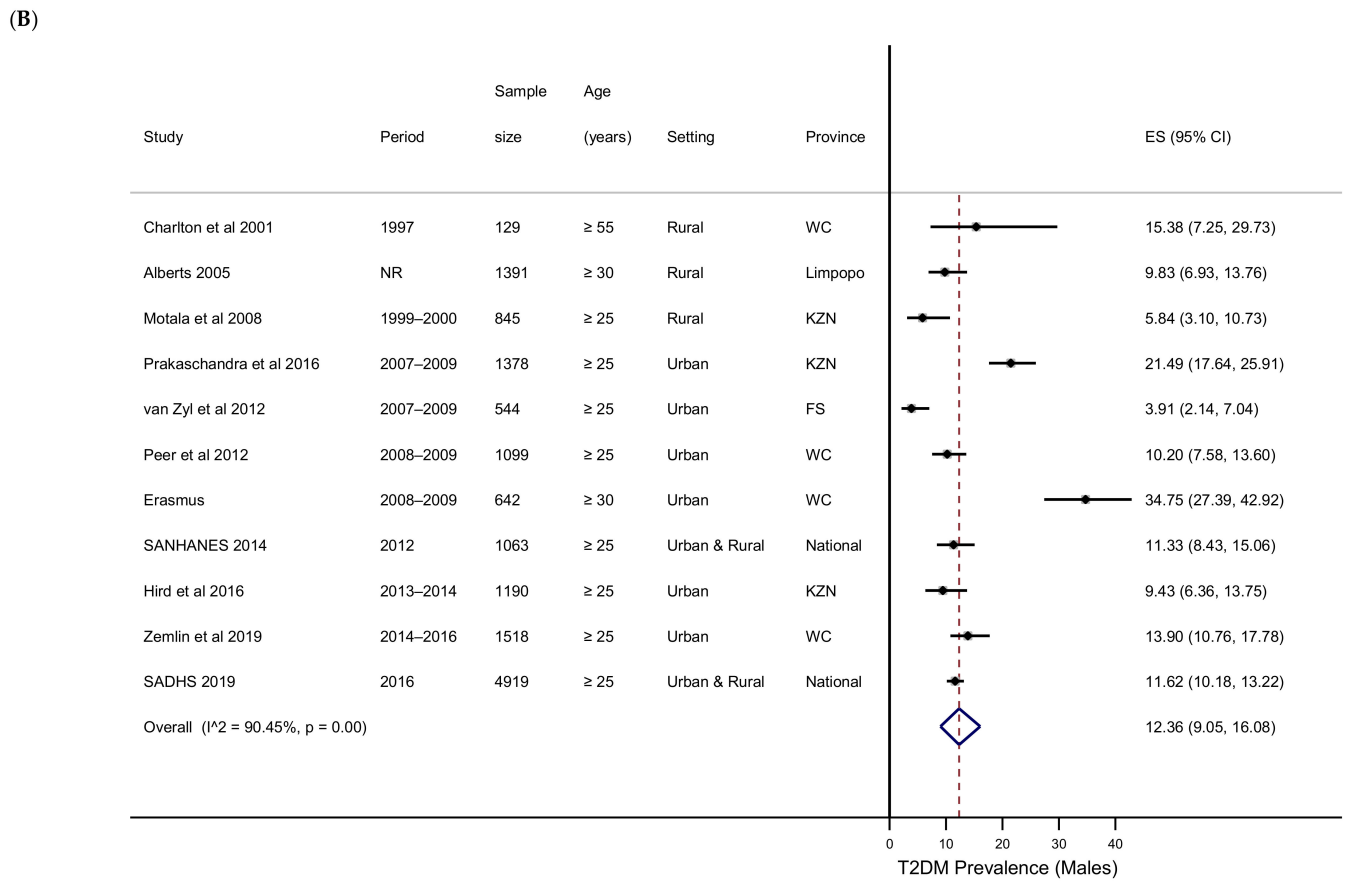
T2DM prevalence in females (**A**) and males (**B**).

**Figure 4:**
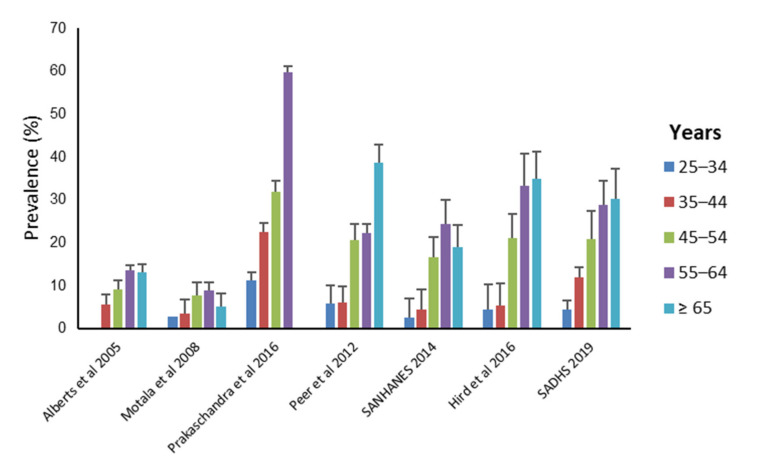
T2DM prevalence across age categories.

Subgroup analysis was conducted to investigate heterogeneity across studies. When stratified by population group, the pooled prevalence in Black Africans was 11.30% (6.97–16.52%), 23.70% (17.13–30.97%) in Coloureds, 6.24% (4.79–7.87%) in a mixed sample of Black Africans and Coloureds, and 14.82% (13.93–15.73%) in the national surveys. The one study that was conducted in Indians, reported a high T2DM prevalence (35.2% (32.60–37.90%)). Significant variation within studies conducted in the Black African population group was observed (95.95%, *p* < 0.001). When stratified by province, the pooled prevalence in FS was 6.24% (4.79–7.87%), 17.36% (3.87–37.71%) in KZN, 21.21% (15.65–27.35%) in the WC and 14.82% (13.93–15.73%) nationally. The one study conducted in Limpopo reported a prevalence 9.94% (8.48–11.62%). A slightly higher pooled prevalence of T2DM was calculated in urban compared to rural settings (18.63% (11.41–27.14%) vs. 10.44% (6.30–15.45%). However, the study that measured prevalence in both settings in the FS, reported a lower prevalence in urban compared to rural populations (4.34% (2.76–6.75%) vs. 7.90 (5.92–10.48%)). Studies used different methods to assess dysglycaemia, making it difficult to compare tests. Three studies directly compared the performance of different diagnostic tests, with varied results. Hird et al. 2016 reported a similar prevalence irrespective whether FPG, 2-h glucose or HbA1c was used [41], while Erasmus et al. 2012 found that FPG was approximately 36% more sensitive than 2-h glucose [12], and Zemlin et al. 2019 demonstrated that 2-h glucose was 30% more sensitive than FPG [42]. Eight studies reported T2DM prevalence by gender. A higher overall prevalence was observed in females compared to males. The pooled estimates in females and males were 16.78% (12.63–21.39%) and 12.36% (9.05–16.08%), respectively (Figure 3). Seven studies reported T2DM prevalence by age group. T2DM prevalence increased across all age groups (Figure 4).

### 3.5. Impaired Glucose Tolerance, Impaired Fasting Glucose and Newly Diagnosed Diabetes

The pooled estimate for IGT was 9.59% (5.82–14.17%), IFG was 3.55% (0.38–9.61%), and newly diagnosed T2DM was 8.29% (4.97–12.34%) (Table 1). As with T2DM prevalence, significant variation across population groups was observed (Table S5).

### 3.6. Publication Bias

We attempted to minimise publication bias in this review in several ways. A comprehensive search, which included consulting with experts to identify grey or unpublished literature, was conducted. Four major databases were searched. Reference lists of articles were screened to identify potentially eligible studies. At least two review authors independently scrutinised and selected articles for inclusion using pre-specified eligibility criteria, assessed risk of bias and extracted data. The funnel plot of the data indicated significant publication bias and heterogeneity (Figure S1).

### 3.7. GRADE

The overall level of evidence as qualified with GRADE was very low as shown in Table S6 due to limitations in study design; poor response rate; unclear risk of bias; methodological limitations; more studies reporting on female population creating gender bias, which negatively affects generalisability; and wide confidence intervals.

## 4. Discussion

This systematic review and meta-analysis pooled prevalence data from 11 population-based studies. The pooled prevalence in individuals 25 years and older was 15.25% (11.07–19.95%) for T2DM, 9.59% (5.82–14.17%) for IGT, 3.55% (0.38–9.61%) for IFG and 8.29% (4.97–12.34%) for newly diagnosed T2DM. Subgroup analysis showed significant heterogeneity between studies in terms of population characteristics, geographical setting, and diagnostic test. T2DM prevalence was higher in females compared to males and increased with age in both sexes.

The greatest source of heterogeneity in T2DM prevalence was population group. In South Africa, population group refers to previously defined apartheid racial categories of Black African, Indian, White (European descent), and Coloured (of mixed ancestry according to the preceding categories), which was introduced into the birth and death notification registries in 1998 to track health inequalities [29]. T2DM prevalence was the highest for Indians, followed by Coloureds, and lowest in Black Africans, which may reflect the state of health transition and non-communicable disease mortality rates in South Africa [43]. Population group differentials are postulated to be due to previous discriminatory practices in South Africa, which has led to disparities in socio-economic status and risk factors for T2DM. Furthermore, biological factors such as genetics [44] and physiological differences such as body fat distribution [45,46] may also contribute to differences in T2DM prevalence between population groups.

T2DM prevalence increased with age, consistent with global epidemiological data [2]. In South Africa, health system improvements in access to care and the widespread implementation and success of antiretroviral (ART) regimens had a significant impact on increasing life expectancy and risk for T2DM [47]. In addition to population aging, urbanisation is also considered a major contributor to T2DM burden in South Africa. Urbanisation is associated with the adoption of unhealthy lifestyles, characterised by the consumption of high-fat and sugary foods and sedentary lifestyles. However, urban/rural disparities in T2DM prevalence were not markedly apparent in our review. These findings are consistent with previous studies comparing T2DM prevalence in urban and rural communities in South Africa and Zambia [48]. Importantly, van Zyl et al. 2012 reported that FPG concentrations ≥ 11.0 mmol/L were almost fourfold higher in women from rural communities compared to those in urban settings [49], highlighting the high T2DM burden in rural communities of South Africa and the need for intervention in these underserved communities. T2DM prevalence was higher in females compared to males, and may be attributed to higher rates of obesity [9] and insulin resistance [50] in South African women compared to men. A study conducted in a population of urban Black African women in Gauteng reported an IGT prevalence of 20.3% [51], identifying these women as a high-risk group that requires monitoring and intervention to prevent the development of T2DM. Furthermore, these women are of reproductive age and at risk for developing gestational diabetes, which poses an intergenerational risk of obesity and T2DM to their children [52].

There are advantages and disadvantages to each diagnostic test for T2DM, with no single preferred test [53]. The OGTT measures glucose tolerance by assessing the body’s response to a 75 g glucose load and is considered the gold standard for T2DM diagnosis. However, the test is cumbersome requiring multiple blood draws and takes at least two hours to perform. The OGTT negatively impacts response rate, thus increasing sampling bias in population surveys. The FPG and HbA1c tests are recommended as an alternative to the OGTT depending on health care facility and resources [54]. Of the three included studies that compared the diagnostic utility of different tests, one reported that 2-h glucose (OGTT) detected 30% more cases [42], another reported that FPG detected 36% more cases [12], while one study showed no significant differences between 2-h glucose and FPG [41]. These findings were surprising since a recent systematic review reported that T2DM prevalence was two-fold higher when using 2-h glucose compared to FPG alone [33]. Differences between populations and disease aetiology may have contributed to the disparities between studies. Despite the similar performances of 2-h glucose and FPG reported in this review, the use of the OGTT is recommended in epidemiological studies to identify those with IGT and for estimating future trends and overall burden of glucose intolerance and T2DM. The HbA1c test does not require fasting and is clinically more convenient and acceptable to patients than the FPG and OGTT. However, although not included in the systematic review due to nongeneralisability, a study conducted in a TB clinic reported that HbA1c overestimated T2DM prevalence ~two-fold [55]. Physiological differences related to red blood-cell turnover (anaemia and iron status), especially in a setting of infection, has been suggested to contribute to regional variations [56]. Thus, despite the advantages HbA1c, poor sensitivity [57] and false positivity in TB patients limit its use in South Africa.

HIV infection and ART exposure has been associated with increased dysglycaemia and T2DM in both community [58] and health care [59] settings in Cape Town. Unfortunately, the included studies did not report prevalence of T2DM by HIV status. Interestingly, two studies from the Limpopo province, one conducted in a hospital, and the other a community-based study, reported T2DM prevalence according to HIV status, with lower rates reported in HIV-positive individuals. While these studies are not directly comparable, similar findings were previously reported in South Africa [48,60], and have been attributed to lower adiposity amongst HIV-positive individuals or improved health awareness. Alternatively, HIV infection could influence T2DM progression and increase severity leading to early mortality, which would translate to decreased prevalence. This hypothesis requires further exploration. Unfortunately, ART data by diabetes status were not reported, thus we are not able to assess whether ART contributed to diabetes risk [61] and is a limitation of prevalence studies in South Africa.

Given the significant heterogeneity observed across studies, the appropriateness of conducting a meta-analysis and calculating a pooled estimate is questionable. Recently, a review of 235 prevalence studies reported that the number of systematic reviews of prevalence data has increased more than ten-fold between 2008 and 2018 and, of these, 65% included a meta-analysis despite significant heterogeneity [23]. These authors conclude that a meta-analysis may be useful to estimate burden of disease, although the use of appropriate statistical models and standardised synthesis and reporting methods is critical. The pooled estimate should be interpreted with caution and in the context of the uncertainty estimates and population heterogeneity.

This was the first systematic review of total and new T2DM, IGT, and IFG prevalence in South Africa. Although we used rigorous systematic review methods, standardised risk-of-bias tools, and PRISMA guidelines, inadequate reporting of methods limited the risk- of-bias and quality assessments of studies, while choice of study population limited the generalisability of the findings. Population characteristics were identified as the greatest source of heterogeneity; however, variable sensitivities and specificities of different diagnostic tests for T2DM may result in over- or underestimation of prevalence. Our review highlights the need for standardised guidelines and reporting methods for prevalence studies that will undoubtedly contribute to calculating a more accurate estimate.

## 5. Conclusions

Although our pooled estimate may be imprecise due to significant heterogeneity between studies and the low level of evidence as assessed by GRADE, we provide evidence that the burden of glucose intolerance (T2DM, IGT, IFG, and newly diagnosed T2DM) in South Africa is high. Findings from this review have significant potential to better inform health policy and planning to better manage this high T2DM disease burden. Our estimates are higher than the 5.4% estimated by the IDF [2], and possibly relate to the exclusion of participants younger than 25 years in our study. Furthermore, we highlight the heterogeneity of T2DM prevalence across population groups, age, gender, setting, and diagnostic test, which all contribute to the challenges of measuring prevalence and leading to the paucity of nationally representative T2DM prevalence data. Well-designed epidemiological studies that use best-practice and standardised methods to assess prevalence are urgently required. Collaboration between public health scientists, diabetes specialists, the community, and policy makers are essential to enable the collection of reliable national epidemiological data to guide health policy and planning.

## Figures and Tables

**Table 1:** Pooled prevalence of T2DM, IGT, IFG, and newly detected T2DM in South Africa.

Condition	Pooled Estimate (95% CI)	I^2^, *p* Value
T2DM (%)	15.25 (11.07–19.95)	98.10%, *p* < 0.001
IGT (%)	9.59 (5.82–14.17)	94.18%, *p* < 0.001
IFG (%)	3.55 (0.38–9.61)	98.68%, *p* < 0.001
Newly detected T2DM (%)	8.29 (4.97–12.34)	94.20%, *p* < 0.001

I^2^ represents the variation in pooled estimate attributable to heterogeneity. *p* represents the level of significance. IFG, impaired glucose tolerance; IGT, impaired glucose tolerance; T2DM, type 2 diabetes mellitus.

## Data Availability

No additional data available.

## Supplementary Materials

The following are available online at https://www.mdpi.com/article/10.3390/ijerph18115868/s1, Table S1: PRISMA 2009 Checklist. Table S2: PubMed search strategy. Table S3: Quality assessment criteria for prevalence studies. Table S4: Prevalence of T2DM in South Africans aged 25 years and older. Table S5: Prevalence of IGT, IFG and undiagnosed T2DM in South Africans aged 25 years and older. Table S6: Level of evidence as qualified with GRADE. Figure S1: Funnel plot of included studies.
