# Supplementary material for: Prevalence of Type 2 Diabetes in South Africa: A Systematic Review and Meta-Analysis
Source: Int J Environ Res Public Health. 2021 May 30;18(11):5868. doi: 10.3390/ijerph18115868 (PMC8199430; doi:10.3390/ijerph18115868)
Supplement: Supplementary file 1 [file ijerph-18-05868-s001.zip › ijerph-1212806-supplementary.pdf]

Prevalence of type 2 diabetes in South Africa: a systematic review

Carmen Pheiffer[1,2*], Victoria Pillay-van Wyk[3], Eunice Turawa[3], Naomi Levitt[4], Andre Kengne[5], Debbie Bradshaw[3,6]

[1] Biomedical Research and Innovation Platform, South African Medical Research Council, Tygerberg, South Africa
[2] Division of Medical Physiology, University of Stellenbosch, Tygerberg, South Africa
[3] Burden of Disease Research Unit, South African Medical Research Council, Tygerberg, South Africa
[4] Division of Endocrinology and Metabolism, University of Cape Town, Observatory, South Africa.
[5]Non-Communicable Diseases Research Unit, South African Medical Research Council, Tygerberg, Western Cape, South Africa
[6] School of Public Health and Family Medicine, University of Cape Town, Observatory, South Africa

*Corresponding author:
Carmen Pheiffer
Biomedical Research and Innovation Platform, South African Medical Research Council
Francie van Zijl Drive, P.O. Box 19070, Tygerberg 7505, South Africa
Tel: +27 21 938 0292
E-mail: carmen.pheiffer@mrc.ac.za

Table S1: *PRISMA 2009 Checklist*

Section/topic	#	Checklist item	Reported on page #
**TITLE**			
Title	1	Identify the report as a systematic review, meta-analysis, or both.	1
**ABSTRACT**			
Structured summary	2	Provide a structured summary including, as applicable: background; objectives; data sources; study eligibility criteria, participants, and interventions; study appraisal and synthesis methods; results; limitations; conclusions and implications of key findings; systematic review registration number.	2
**INTRODUCTION**			
Rationale	3	Describe the rationale for the review in the context of what is already known.	4-5
Objectives	4	Provide an explicit statement of questions being addressed with reference to participants, interventions, comparisons, outcomes, and study design (PICOS).	5
**METHODS**			
Protocol and registration	5	Indicate if a review protocol exists, if and where it can be accessed (e.g., Web address), and, if available, provide registration information including registration number.	6
Eligibility criteria	6	Specify study characteristics (e.g., PICOS, length of follow-up) and report characteristics (e.g., years considered, language, publication status) used as criteria for eligibility, giving rationale.	6-7

**Information sources**	**7**	**Describe all information sources (e.g., databases with dates of coverage, contact with study authors to identify additional studies) in the search and date last searched.**	**6**
**Search**	**8**	**Present full electronic search strategy for at least one database, including any limits used, such that it could be repeated.**	**6, Supplementary Table 1**
**Study selection**	**9**	**State the process for selecting studies (i.e., screening, eligibility, included in systematic review, and, if applicable, included in the meta-analysis).**	**7-8**
**Data collection process**	**10**	**Describe method of data extraction from reports (e.g., piloted forms, independently, in duplicate) and any processes for obtaining and confirming data from investigators.**	**8**
**Data items**	**11**	**List and define all variables for which data were sought (e.g., PICOS, funding sources) and any assumptions and simplifications made.**	**8**
**Risk of bias in individual studies**	**12**	**Describe methods used for assessing risk of bias of individual studies (including specification of whether this was done at the study or outcome level), and how this information is to be used in any data synthesis.**	**7-8**
**Summary measures**	**13**	**State the principal summary measures (e.g., risk ratio, difference in means).**	**7**
**Synthesis of results**	**14**	**Describe the methods of handling data and combining results of studies, if done, including measures of consistency (e.g., I^2) for each meta-analysis.**	**8-9**
**Risk of bias across studies**	**15**	**Specify any assessment of risk of bias that may affect the cumulative evidence (e.g., publication bias, selective reporting within studies).**	**9**
**Additional analyses**	**16**	**Describe methods of additional analyses (e.g., sensitivity or subgroup analyses, meta-regression), if done, indicating which were pre-specified.**	**9**

**RESULTS**			
Study selection	17	Give numbers of studies screened, assessed for eligibility, and included in the review, with reasons for exclusions at each stage, ideally with a flow diagram.	10, Figure 1
Study characteristics	18	For each study, present characteristics for which data were extracted (e.g., study size, PICOS, follow-up period) and provide the citations.	10
Risk of bias within studies	19	Present data on risk of bias of each study and, if available, any outcome level assessment (see item 12).	10, Supplementary Table 3
Results of individual studies	20	For all outcomes considered (benefits or harms), present, for each study: (a) simple summary data for each intervention group (b) effect estimates and confidence intervals, ideally with a forest plot.	11, Figures 2,3
Synthesis of results	21	Present results of each meta-analysis done, including confidence intervals and measures of consistency.	24, Table 1
Risk of bias across studies	22	Present results of any assessment of risk of bias across studies (see Item 15).	13, Supplementary Table 5
Additional analysis	23	Give results of additional analyses, if done (e.g., sensitivity or subgroup analyses, meta-regression [see Item 16]).	11
**DISCUSSION**			
Summary of evidence	24	Summarize the main findings including the strength of evidence for each main outcome; consider their relevance to key groups (e.g., healthcare providers, users, and policy makers).	14

**Limitations**	**25**	**Discuss limitations at study and outcome level (e.g., risk of bias), and at review-level (e.g., incomplete retrieval of identified research, reporting bias).**	**3, 17**
**Conclusions**	**26**	**Provide a general interpretation of the results in the context of other evidence, and implications for future research.**	**3, 17**
**FUNDING**			
**Funding**	**27**	**Describe sources of funding for the systematic review and other support (e.g., supply of data); role of funders for the systematic review.**	**18**

From: **Moher D, Liberati A, Tetzlaff J, Altman DG, The PRISMA Group (2009). Preferred Reporting Items for Systematic Reviews and Meta-Analyses: The PRISMA Statement. PLoS Med 6(6): e1000097. doi:10.1371/journal.pmed1000097**

Table S2. *PubMed search strategy*

Search	Query
#4	Search ((#3 NOT (animals[mh] NOT humans[mh]))) AND (“1997/01/01”[Date-Publication] : “2020/06/30”[Date-Publication])
#3	Search (#1 AND #2)
#2	Search (South Africa[mh]OR“South Africa*”[tiab] OR RSA[tiab] OR Africa, Southern[mh:noexp] OR Southern Africa[tiab])
#1	Search (Diabetes[Mesh] OR Diabetes mellitus[Mesh] OR type 2 diabetes mellitus[Mesh] OR type 2 diabetes[Mesh] OR Diabetes mellitus, type 2[Mesh] OR Diabetes, type 2[Mesh] OR hyperglycemia[Mesh] OR blood glucose[Mesh] OR Hemoglobin A, glycosylated[Mesh] OR Glycosylated hemoglobin OR diagnosis OR impaired glucose tolerance OR impaired fasting glucose OR undiagnosed diabetes

The PubMed search strategy was adapted for optimal searching in the other databases.

Table S3. *Quality assessment criteria for prevalence studies*

Domain	Criteria	Question	Score
External validity	Representativeness	Was a sample size calculation conducted and is it adequate?	1
		Is the target population a close representation of the national population in relation to relevant variables?	1
		Was the sampling frame a true or close representation of the population?	1
		Was a form of random selection used to select the sample? Was the sampling method appropriate for the research question?	2
	Non-response bias	Were there similarities between participants and non-participants in relation to demographic characteristics?	1
		Was the overall/response rate of the study reported?	1
		What was the overall/response rate for the study?	1
		Was the overall/response rate adequate for the study? Excellent ≥80%, Average 60-79%, Poor <60%	1
Internal validity	Case definition	Were the cases classified using the ICD codes or was an acceptable case definition used? What is the case definition?	1
		Were the study instruments used to measure the parameter of interest shown to have reliability and validity in this study or a previous study?	2
	Data collection	Were data collected directly from the participants or is a proxy was used, was it appropriate?	1
		Was the same mode of date collection used for all participants for the condition of interest?	1
	Uncertainty of estimation	Was the parameter of interest reported with uncertainty, i.e. Standard deviation (SD), Standard Error (SE) or 95% Confidence Interval (CI)?	1
	Appropriateness of time factor for outcome measure	Was the length of recall period for the parameter of interest appropriate to ascertain outcome/exposure?	2
	Appropriateness of numerator and denominator in calculation of estimate	Were the numerator and the denominator for the parameter of interest appropriate? If not, can these be extracted to recalculate the parameter of interest?	2
	Confounding	Were potential confounding factors sought and controlled for?	1
		Total Score	20

Risk of bias was assessed using a web-based standardised checklist for systematic review of observational epidemiological studies, Burden of Disease Review Manager (BODRevMan) developed by the South African Medical Research Council [31], that was adapted from the risk of bias tool for population-based studies [36] and the Newcastle-Ottawa Scale for assessing the quality of non-randomised studies [37,38].

Table S4. *Prevalence of T2DM in South Africans aged 25 years and older.*

Author	Province	Sample size	Year	Population group	Age (years)	T2DM Prevalence									Test	Diagnostic criteria	Risk of bias
						Urban			Rural			Urban and Rural					
						Female	Male	Total	Female	Male	Total	Female	Male	Total			
Charlton et al 2001 [44]	WC	152	1997	Coloured	≥ 55				28.9 (19.5-38.2)	15.8 (4.2-27.4)	24.6 (17.2-32)				OGTT	WHO, 1985	Moderate
Alberts et al 2005 [45]	Limpopo	1391	NR	Black African	≥ 30				10.0 (8.3-12.0)	9.9 (6.7-13.8)	9.9 (8.4-11.6)				FPG	WHO, 1998	Moderate
Motala et al 2008 [26]	KZN	1025	1999-2000	Black African	≥ 25				5.4 (3.8-7.3)	5.8 (2.7-6.6)	5.4 (4.0-7.2)				FPG/ OGTT	WHO,1998	Low
Prakaschandra et al 2016 [42]	KZN	1428	2007-2008	Indian	25-64			35.2 (32.6-37.9)							FPG	WHO, 2006	Low
van Zyl et al 2012 [47]	FS	955	2007-2009	Black African & Coloured	25-64	5.1 (2.9-8.1)	2.1 (0.3-7.3)	4.3 (2.6-6.8)	9.1 (6.4-12.4)	5.0 (2.2-9.6)	7.9 (5.8-10.5)				FPG	WHO, 1998	Low
Peer et al 2012 [16]	WC	1099	2008-2009	Black African	25-74	13.8 (11.4-16.3)	10.2 (7.1-13.4)	12.1 (10.2-14.0)							FPG/ OGTT	WHO, 1998	Low
Erasmus et al 2012 [17]	WC	642	2008-2009	Coloured	≥ 30			28.2 (24.6-32.2)							FPG/ OGTT	WHO, 2008	Low
SANHANES 2014 [21]	National	1063	2012	All	≥ 25							17.7 (13.5-22.8)	11.3 (7.3-17.0)	14.7 (11.8-18.3)	HbA1c	WHO,2011	Moderate
Hird et al 2016 [46]	KZN	1190	2013- 2014	Black African	≥ 25	19.1 (16.1-22.3)	9.4 (6.1-13.8)	16.5 (14.1-19.0)							FPG/ OGTT	WHO, 1998, 2011	Low
Zemlin et al 2019 [43]	WC	1518	2014-2016	Coloured	≥ 30	20.9 (18.6-23.4)	13.9 (10.6-17.8)	19.1 (17.2-21.2)							FPG/ OGTT	WHO, 2006	Moderate
SADHS 2019 [21]	National	4919	2016	All	≥ 25							17.3 (15.7-19.1)	11.6 (9.9-13.6)	14.9 (13.6-16.3)	HbA1c	WHO, 2011	Moderate

NR, not reported. EC, Eastern Cape; KZN, KwaZulu Natal; WC, Western Cape.

Table S5. *Prevalence of IGT, IFG and undiagnosed T2DM in South Africans aged 25 years and older.*

Author	Province	Sample size	Year	Population group	Age (years)	IGT Prevalence		IFG Prevalence		Undiagnosed T2DM		Test	Diagnostic criteria
						Urban	Rural	Urban	Rural	Urban	Rural		
Charlton et al 2001 [44]	WC	152	1997	Coloured	≥ 55		11.5 (5.9-17.0)					OGTT	WHO, 1985
Motala et al 2008 [26]	KZN	1025	1999-2000	Black African	≥ 25		7.5 (5.8-9.4)		1.4 (0.7-2.5)		4.6 (3.3-6.3)	FPG/ OGTT	WHO,1998
Prakaschandra et al 2016 [42]	KZN	1428	2007-2008	Indian	25-64	15.6 (13.7-17.8)		31.4 (28.8-34.0)				FPG	WHO, 2006
Peer et al 2012 [16]	WC	1099	2008-2009	Black African	25-74	10.7 (8.9-12.6)		1.2 (0.6-1.9)		4.9 (3.7-6.3)		FPG/ OGTT	WHO, 1998
Erasmus et al 2012 [17]	WC	642	2008-2009	Coloured	≥ 30	15.3 (12.4-18.5)		4.4 (2.9-6.5)		18.1 (15.0-21.6)		FPG/ OGTT	WHO, 2008
Hird et al 2016 [46]	KZN	1190	2013- 2014	Black African	≥ 25	4.3 (3.1-5.8)		0.9 (0.4-1.7)				FPG/ OGTT	WHO, 1998, 2011
Zemlin et al 2019 [43]	WC	1518	2014-2016	Coloured	≥ 30					6.3 (5.1-7.6)		FPG/ OGTT	WHO, 2006

EC, Eastern Cape; KZN, KwaZulu Natal; WC, Western Cape.

Table S6. *Level of evidence as qualified with GRADE*

Certainty assessment							№ of patients	Prevalence estimates	Certainty
№ of studies	Study design	Risk of bias	Inconsistency	Indirectness	Imprecision	Other considerations	T2DM	(%)	
Prevalence of Type 2 Diabetes (assessed with: The following criteria was used to diagnosed type 2 diabetes: 1. WHO (2006) diagnostic criteria where type 2 diabetes is diagnosed either by a physician, fasting blood glucose concentrations ≥7.0 mmol/L, 2-hour oral glucose tolerance test values ≥11.1 mmol/L or self-reported use of oral diabetes drugs. 2. Glycated haemoglobin ≥6.5% (48 mmol/mol))									
11	observational studies	serious [a]	serious [b]	serious [c]	serious	none	14,685	15.25 (11.07-19.95)	⊕◯◯◯ [abc] VERY LOW
Impaired glucose tolerance (IGT) (assessed with: IGT measured using FPG <7.0 mmol/L and 2-hour OGTT plasma ≥7.8 mmol/L and <11.1 mmol/L)									
5	observational studies	serious [a]	serious [b]	not serious	serious [a]	none	3,592	9.59 (5.82-14.17)	⊕◯◯◯ [ab] VERY LOW
Undiagnosed Type 2 Diabetes (assessed with: • T2DM defined as fasting plasma glucose (FPG) ≥7.0 mmol/L, 2-hour oral glucose tolerance test (OGTT) plasma glucose ≥11.1 mmol/L, glycated haemoglobin (HbA1c) ≥6.5% (48 mmol/mol))									
7	observational studies	serious [a]	serious [b]	not serious	serious [a]	none	4,205	8.29 (4.97-12.34)	⊕◯◯◯ [ab] VERY LOW
Impaired fasting glucose (assessed with: Assessed using >6.1mml/L and <7.0 mml/L)									
5	observational studies	serious [a]	serious [b]	not serious	serious [b]	none	4,710	3.55 (0.38-9.61)	⊕◯◯◯ [ab] VERY LOW

CI: Confidence interval

Explanations

a. Downgraded by 1 because of limitations in studies design, poor response rate and unclear of risk of bias.

b. Downgraded by 1 because of methodological limitations

c. More studies reporting on female population creating gender bias which negatively affects generalizability

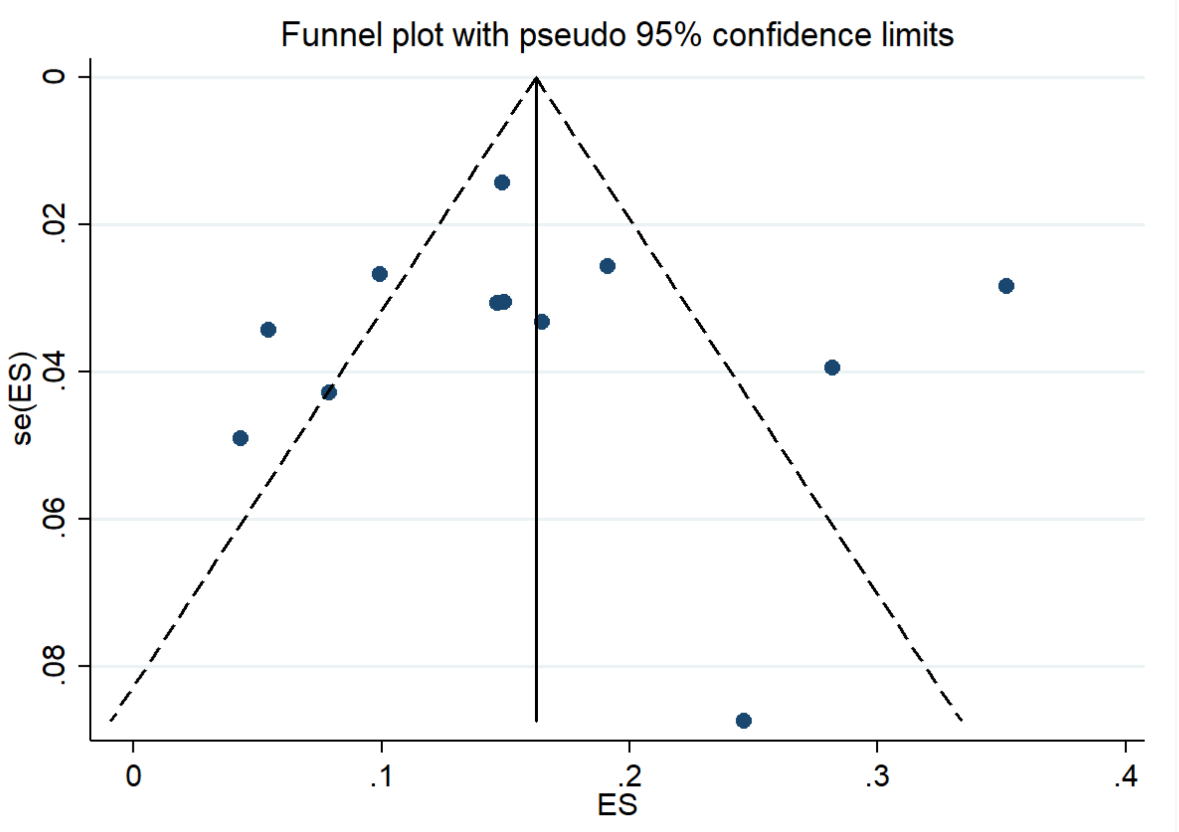

Figure S1. *Funnel plot of included studies.*
